# Preparation of Sn-Doped Ga_2_O_3_ Thin Films and MSM Ultraviolet Detectors Using Magnetron Co-Sputtering

**DOI:** 10.3390/ma17133227

**Published:** 2024-07-01

**Authors:** Yantao Liu, Rong Huang, Tao Lin, Jiale Dang, Haoxiang Huang, Jiahao Shi, Sui Chen

**Affiliations:** Department of Electronic Engineering, Xi’an University of Technology, Xi’an 710048, Chinalintao@xaut.edu.cn (T.L.); 2220321293@stu.xaut.edu.cn (J.D.); 1397187832@stu.xaut.edu.cn (H.H.);

**Keywords:** Ga_2_O_3_ thin film, Sn-doped, photoelectric, ultraviolet detector

## Abstract

Sn-doped Ga_2_O_3_ thin films and metal–semiconductor–metal (MSM) ultraviolet detectors were prepared using the co-sputtering method to enhance their photoelectric performance. The results revealed that Sn doping can effectively change the optical and electrical properties of thin films, greatly improving the photoelectric responsiveness of the devices. Through microstructure testing results, all of the thin film structures were determined to be monoclinic beta phase gallium oxide. At a DC power of 30 W, the thickness of the Sn-doped thin film was 430 nm, the surface roughness of the thin film was 4.94 nm, and the carrier concentration, resistivity, and mobility reached 9.72 × 10^18^ cm^−3^, 1.60 × 10^−4^ Ω·cm, and 45.05 cm^3^/Vs, respectively. The optical results show that Sn doping clearly decreases the transmission of thin films and that the bandgap can decrease to 3.91 eV. Under 30 W DC power, the photo dark current ratio of the detector can reach 101, time responses of *t_r_* = 31 s and *t_f_* = 22.83 s were obtained, and the spectral responsivity reached 19.25 A/W.

## 1. Introduction

UV detectors are widely used in military and civilian fields such as solar ultraviolet detection, astronomy, space security communication, optical imaging, detection of flames/arc light, UV disinfection, and various types of environmental monitoring due to their low background noise, high sensitivity, and strong anti-interference ability, which can effectively reduce false alarm rates and reduce signal processing difficulties [1,2,3,4]. As a semiconductor material with a direct bandgap corresponding to the deep ultraviolet band and an ultra-wide bandgap (~4.9 eV), gallium oxide (Ga_2_O_3_) thin films have a high breakdown electric field, excellent thermal and chemical stability, high UV visible transmittance, and high radiation resistance. Therefore, it is a natural material for preparing day-blind ultraviolet detectors.

During the preparation process of Ga_2_O_3_ thin films, point defects such as oxygen vacancies, Ga vacancies, or Ga interstitial atoms are often introduced [5,6]. Therefore, unintentionally doped Ga_2_O_3_ thin films typically exhibit n-type semiconductor characteristics. However, due to the low carrier concentration and electron mobility in undoped Ga_2_O_3_ films, they typically exhibit poor conductivity, which limits their application in high-performance optoelectronic semiconductor devices, such as devices that typically exhibit lower spectral responsivity, detection rates, and signal-to-noise ratios. However, the photoelectric properties and the bandgap of Ga_2_O_3_ thin films can be adjusted with doped elements, such as Si [7], Sn [8], Ta [9], Ge [10], Zn [11], Mg [12], N [13], Nb [14], and In [15,16]. Ken Goto et al. [7] prepared Si-doped Ga_2_O_3_ films with the carrier density of the films controlled in the range of 3.18 × 10^15^ to 1.18 × 10^18^ cm^−3^ by changing the doping concentration, which changed the resistivity from 13.2 Ω·cm to 0.06 Ω·cm and the activation energy from 44.7 meV to 16.7 meV. Wei Mi et al. [8] prepared Sn-doped Ga_2_O_3_ films with different Sn doping contents on MgAl_2_O_4_ (100) substrates using the metal organic chemical vapor deposition (MOCVD) method. The resistivity of a thin film sample with 10% Sn doping content decreased by about 11 orders of magnitude compared with undoped Ga_2_O_3_ films. Anoop Kumar Singh et al. [12] prepared Zn-doped Ga_2_O_3_ films, with the doped films exhibiting a positive Hall coefficient and the addition of Zn transforming the thin film from an n-type to a p-type. H. Zhang et al. [15] investigated the effect of Nb doping on the oxygen vacancy concentration and optical properties of Ga_2_O_3_ thin films. They found that due to the doping of Nb^5+^, the oxygen vacancy ratio of the thin films deposited at a sputtering power of 5 W on a Nb_2_O_5_ target increased from 1.55 without doping to 1.64. As the Nb-doping content increases, the optical bandgap of the thin film decreased from 5.04 eV to 4.93 eV. Tao Lin et al. [16] prepared In-doped Ga_2_O_3_ thin films with different In-doping contents using the magnetron co-sputtering method; by changing the In component, the transmittance in the visible light range was greater than 95%, and the bandgap width of the film was in the range of 4.94~3.42 eV.

Moreover, by introducing shallow donor impurities into Ga_2_O_3_ thin films, the bandgap width and conductivity of Ga_2_O_3_ thin films can be effectively adjusted, thereby improving the device performance of gallium-oxide-based day-blind ultraviolet detectors [17,18,19,20]. Ming Ming Fan et al. [21] prepared Sn-doped Ga_2_O_3_ thin films with different Sn-doping contents using a chemical vapor deposition method and prepared metal–semiconductor–metal (MSM) type ultraviolet photodetectors based on the prepared thin film samples. By adjusting the Sn-doping content, the optical bandgap of the thin film changed from 5.7 eV to 5.2 eV, corresponding to a red shift of −3 dB at the cutoff edge of the ultraviolet detector, and the response increased from 0.2 mA/W to 80 mA/W. Xi Zhu et al. [22] made InZn-Ga_2_O_3_ nanowires on a Si/SiO_2_ substrate; the doping of In and Zn enhanced the structural stability and conductivity of the films. When the molar ratios of In, Ga, and Zn were 1:2:1, the highest responsivity, 440.42 A/W under 254 nm at 10 V, was obtained, which is 1.19 ×10^4^ times higher than that of pure Ga_2_O_3_ nanowires.

At present, the reported methods for preparing Ga_2_O_3_ films include MOCVD [23], molecular beam epitaxy (MBE) [24], sol gel [25], atomic layer deposition (ALD) [26], pulsed laser deposition (PLD) [27], and radio frequency magnetron sputtering (RFMS) [28]. Jiang Yu et al. [29] used the PLD method to grow a β-Ga_2_O_3_/4H SiC heterojunction, which was subsequently used to prepare optimized ultraviolet photodetectors. The post-annealing temperature during the preparation of the Ga_2_O_3_/4H SiC heterojunction improved the interface defects of the heterojunction, resulting in a high-performance UV detector with ultra-low dark current (0.167 pA), a high light-to-dark suppression ratio (384.3), and a wide linear dynamic range (51.7 dB). Teng Jiao et al. [30] prepared vertical Schottky barrier photodiodes using Ga_2_O_3_ homoepitaxial thin films grown using the MOCVD method. Compared with photodiodes based on heteroepitaxial Ga_2_O_3_ thin films, the devices exhibited higher comprehensive performance. Under a 0 V bias, the response speed was less than 10 ms, R was 0.59 A/W, and Ip/Id was >10^3^. Meng Qiu Li et al. [31] prepared an Al-doped detector using magnetron sputtering on Ga_2_O_3_ thin films grown on SiC substrates to produce a β-Ga_2_O_3_/Al-type ultraviolet detector and investigated the effect of annealing temperature on the material properties and device performance. Under a bias voltage of 10 V, the 800 °C annealed sample was found to exhibit the best photoelectric detection performance for 254 nm ultraviolet light. The device R was 2.6 A/W, the quantum efficiency was 1265%, and the device response time was as low as 0.26 s.

In this article, we use magnetron co-sputtering technology to prepare Sn-doped Ga_2_O_3_ thin films with different Sn doping contents on sapphire substrates by adjusting the sputtering power of SnO_2_ target materials. By using co-sputtering technology to adjust the Sn content, the microstructure and optical properties of Ga_2_O_3_ thin films can be achieved, and ultimately, the performance of MSM ultraviolet detectors can be adjusted based on this foundation. The influence of Sn-doping content on the optoelectronic properties and crystal quality of Ga_2_O_3_ thin films is systematically studied, and the Sn-doping process of Ga_2_O_3_ thin films is optimized. Based on this, an MSM is prepared using DC magnetron sputtering technology, and a systematic study is conducted on the effect of Sn doping on the performance of Sn-doped Ga_2_O_3_-based ultraviolet detectors.

## 2. Materials and Methods

In this experiment, a magnetron sputtering instrument (SP3-80C, Chuangshiweina Technology Co., Beijing, China) was used to prepare Sn-doped Ga_2_O_3_ films with different Sn-doping contents on sapphire substrates using co-sputtering technology. A Ga_2_O_3_ ceramic target with a purity of 99.99% and a SnO_2_ ceramic target (Deyang Ona New Materials Co., Ltd., Deyang, China) with a purity of 99.99% were used as Ga and Sn sources, respectively. The Ga_2_O_3_ target was connected to an RF power supply, while the SnO_2_ target was connected to a DC power supply. To adjust the Sn-doping content in the Sn-doped Ga_2_O_3_ films, the sputtering power of the fixed target Ga_2_O_3_ material was set to 100 W, and the sputtering power of the SnO_2_ target material was adjusted to vary within the range of 0 W to 30 W, the corresponding power density is 0 W/cm^2^, 0.2 W/cm^2^, 0.4 W/cm^2^, and 0.6 W/cm^2^. Before the thin film deposition, the sapphire substrate was ultrasonically cleaned with acetone, deionized water, and anhydrous ethanol for 10 min. After the substrate cleaning was completed, it was blown dry with a nitrogen gun. Then, the dried substrate was heated on a 100 °C constant temperature heating table to remove residual deionized water on the substrate surface. After the substrate cleaning was completed, the sample was placed in a sputtering chamber for sputtering. During the thin film deposition process, the background vacuum degree of the sputtering chamber was set to below 9 × 10^−3^ Pa to avoid contamination by impure gases. Then, argon (Ar) and oxygen (O_2_) with purities of 99.99% and gas flow rates of 81 sccm and 9 sccm were sent into the sputtering chamber as working gases. The sputtering pressure was fixed at 2.5 Pa, the sputtering temperature was room temperature, and the film deposition time was 5 h. The thickness of the Sn-doped Ga_2_O_3_ films was monitored by a film thickness monitor (TM-106A, HF-Kjing Company, Beijing, China). To ensure uniform thickness of the deposited film, the substrate rotation speed was set to 14 rpm. After the thin film deposition was completed, the prepared thin film samples were annealed at 800 °C and in a nitrogen atmosphere for 2 h in a tube-type high-temperature annealing furnace (OTF-1200X, Hefei Kejing Material Technology Co., Ltd., Hefei, China) to complete the high-temperature recrystallization treatment of the Sn-doped Ga_2_O_3_ thin films.

**Figure 1:**
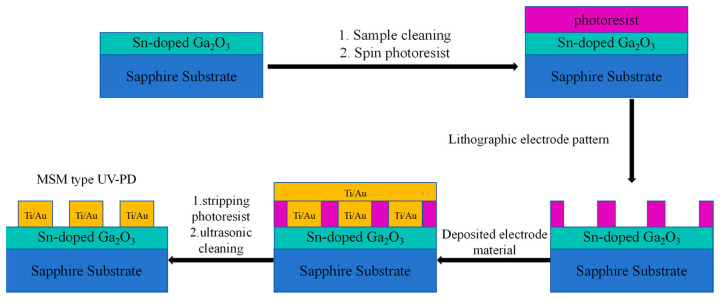
Process flow diagram for Sn-doped Ga_2_O_3_ MSM detector preparation.

**Figure 2:**
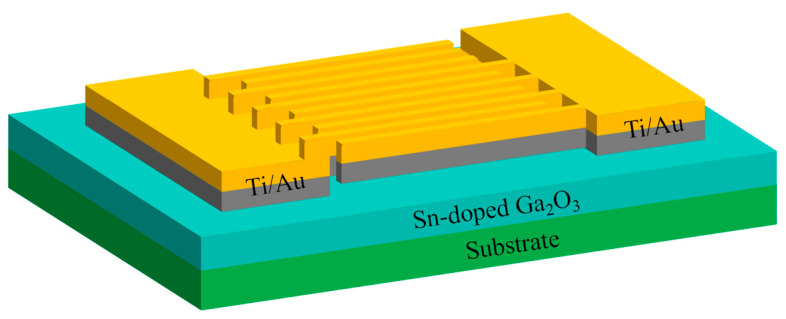
A schematic of the Sn-doped Ga_2_O_3_ MSM detector.

After film preparation, MSM type ultraviolet detectors with cross-finger-shaped electrodes were prepared using the Sn-doped Ga_2_O_3_ films. The device preparation process is shown in Figure 1, which includes the photolithographic cleaning of the film samples, the photolithography of the electrode patterns, the DC magnetron sputtering deposition of Ti/Au electrodes, and de-lamination. A schematic diagram of the device structure is shown in Figure 2, with a fork width of 50 μm. The length of the fork is 500 μm, the interdigital spacing is 20 μm, and the number of cross finger pairs is 5.

**Figure 3:**
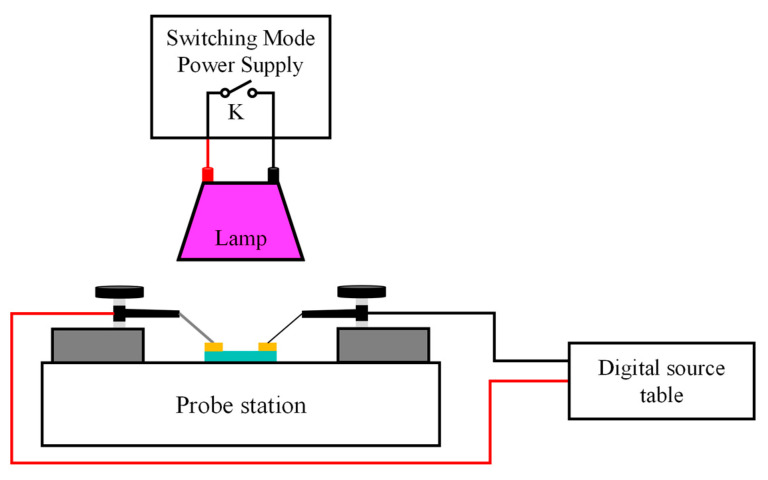
The schematic diagram of MSM detector test system.

The crystal structure of the thin film samples with different Sn-doping contents was characterized using X-ray diffraction (XRD; Smartlab, Rigaku, Tokyo, Japan); the cross-sectional images and Sn-doping contents were tested with a field emission scanning electron microscope (Sigma360, Zeiss, Oberkochen, Germany) equipped with an energy spectrometer; the surface morphology of the thin film samples was characterized using atomic force microscopy (Dimension Icon, Bruker, Billerica, MA, USA); and the optical and electrical properties of the Sn-doped Ga_2_O_3_ thin films were characterized using a Hall effect system and UV visible spectrophotometer (CH300, CH-Magnetoelectricity Technology, China, and Lamda950, PerkinElmer, Waltham, MA, USA, respectively). To analyze the effect of Sn-doping content on the performance of Sn-doped Ga_2_O_3_-based MSM ultraviolet detector devices, a digital source meter (KEITHLEY 2400, Tektronix, Beaverton, OR, USA) with a 254 nm wavelength UV light source and an incident optical power of 100 μW/cm^2^ was used to characterize and analyze the *I–V* characteristics, as shown in Figure 3, and the illumination intensity of the ultraviolet light source was measured using an ultraviolet illuminometer (ST512, SENTRY, New Taipei City, Taiwan).

## 3. Results and Discussion

### 3.1. Microstructure and Component of Sn-Doped Ga_2_O_3_ Thin Films

**Figure 4:**
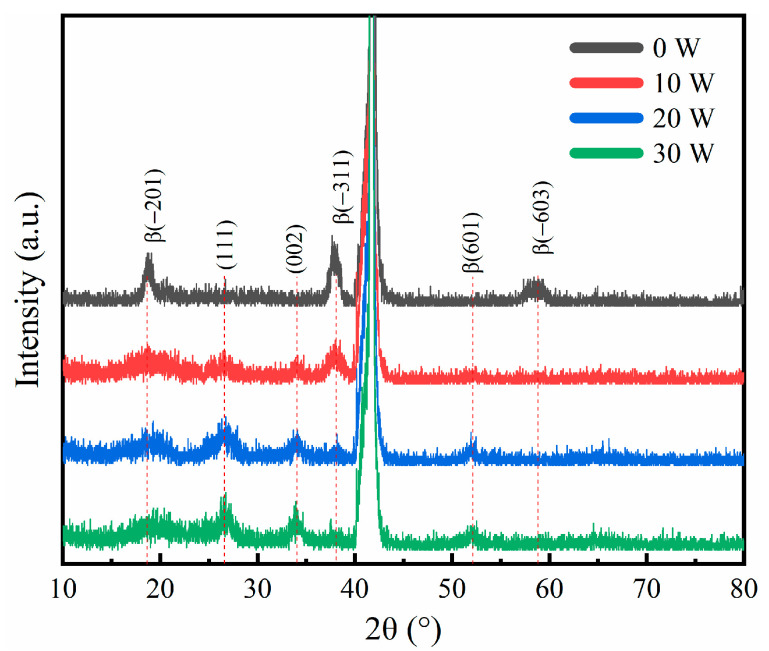
XRD patterns of thin films deposited under different sputtering powers of SnO_2_ target materials.

Figure 4 shows the XRD patterns of the Sn-doped Ga_2_O_3_ films deposited on SnO_2_ target materials with sputtering powers of 0 W, 10 W, 20 W, and 30 W. The diffraction peak with the highest intensity is the (006) diffraction peak on the sapphire substrate. According to the standard JPCDS card of β-Ga_2_O_3_ (PDF #43-1012), an undoped thin film sample and Sn-doped thin films both have monoclinic phases that appear at angles of 18.95° and 38.40°, respectively, and (−201) and (−311) diffraction peaks on the crystal plane [32,33]. With the increase in Sn-doping content, the Sn-doped thin film sample (−311) diffraction peak intensity gradually weakens. With the introduction of Sn doping, the diffraction peak (−603) disappears and the (601), (111), and (002) diffraction peaks appear; this indicates that the Ga^3+^ in the film is replaced by Sn^4+^ [34], and the doping of Sn atoms into the Ga_2_O_3_ lattice leads to a transformation in the film’s crystal structure. As the sputtering power of the SnO_2_ target material increases, the intensity of the diffraction peak in the (−311) crystal direction gradually weakens, with the diffraction peaks corresponding to the crystal orientations of (601), (111), and (002) gradually increasing, indicating that as the Sn doping amount increases, the preferred crystal orientation deteriorates.

**Figure 5:**
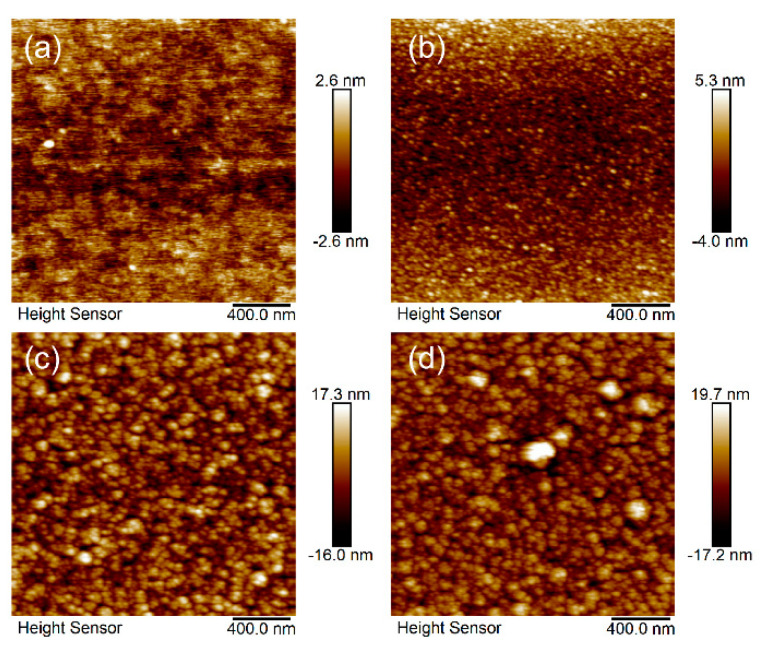
AFM images of Sn-doped Ga_2_O_3_ thin films with different DC power, (**a**) 0 W, (**b**) 10 W, (**c**) 20 W, (**d**) 30 W.

**Figure 6:**
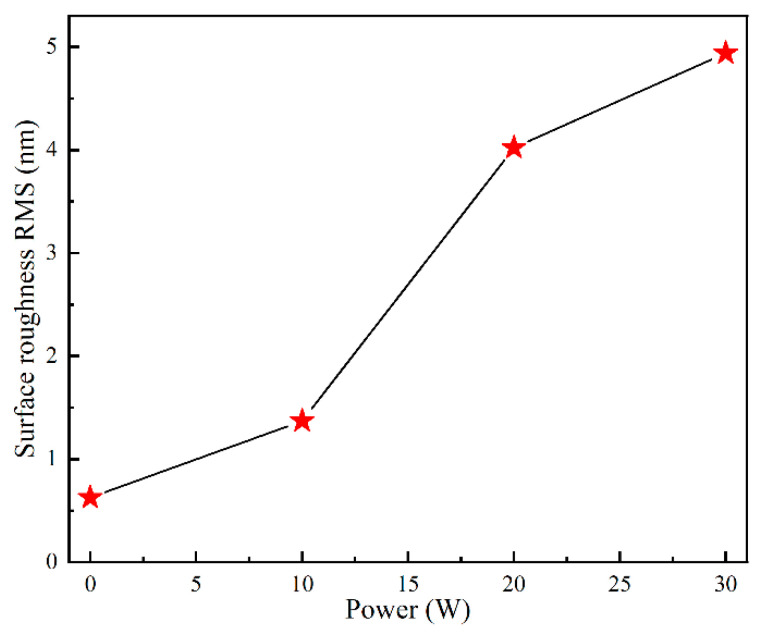
The variation in the surface roughness of thin films with the sputtering power of the SnO_2_ target.

**Figure 7:**
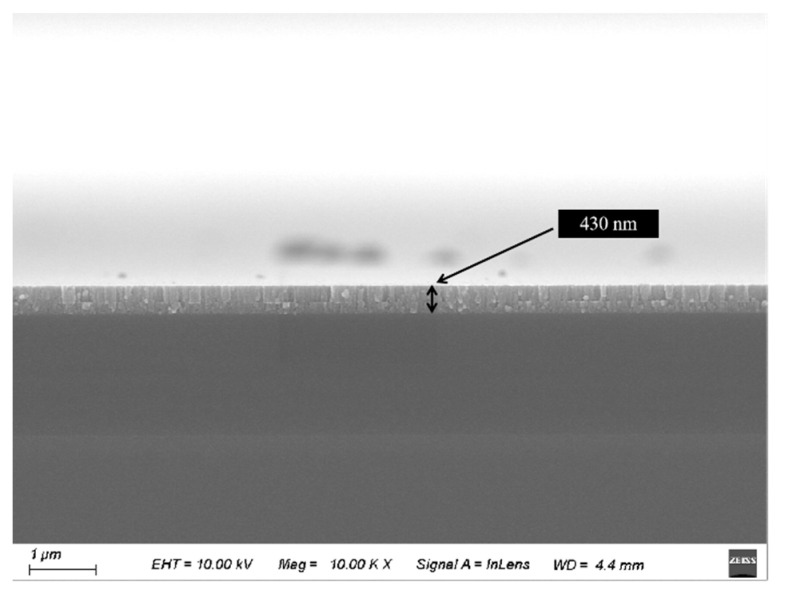
The cross-sectional view of a Sn-doped Ga_2_O_3_ thin film under 30 W DC power.

The surface morphology results of the thin films are shown in Figure 5, while the surface roughness of the thin films is shown in Figure 6. The thickness of the thin film is about 430 nm from Figure 7. The root mean square roughness Rq of the Sn-doped Ga_2_O_3_ thin films deposited at sputtering powers of 0 W, 10 W, 20 W, and 30 W for the SnO_2_ target materials are 0.62 nm, 1.37 nm, 4.02 nm, and 4.94 nm, respectively. This indicates that as the Sn doping content increases, the surface roughness of the thin film increases, and as the sputtering power increases to 30 W, a certain clustering phenomenon occurs in the surface grains of the thin film. The change in surface morphology of the gallium oxide thin films is mainly due to the slightly larger ionic radius of Sn^4+^. Therefore, the introduction of Sn doping leads to a change in the crystalline quality of the thin films.

**Figure 8:**
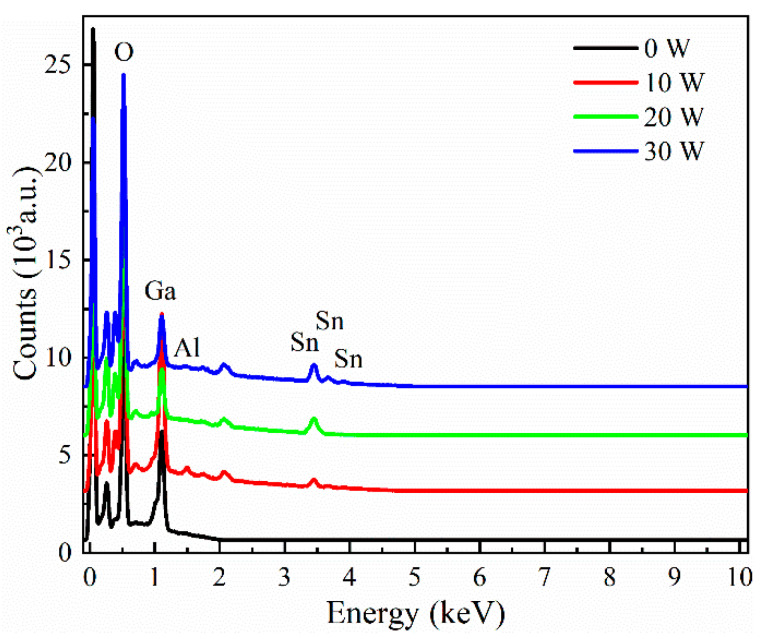
EDS spectra of Sn-doped Ga_2_O_3_ thin films under different sputtering powers of SnO_2_ target materials.

**Figure 9:**
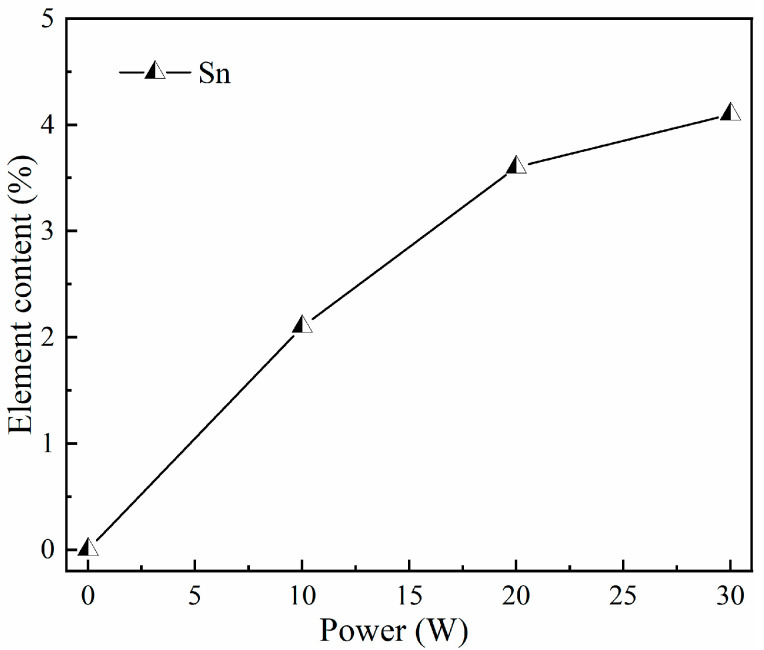
Percentage of Sn atoms at different sputtering powers of SnO_2_ target materials.

The energy dispersive X-ray spectroscopic (EDS) results of the Sn-doped Ga_2_O_3_ thin films are shown in Figure 8. As the sputtering power of the SnO_2_ target material increases from 0 W to 30 W, the Sn-doping content in the film significantly increases, which can be attributed to the effective substitution of Ga atoms by Sn during the film deposition process. As shown in Figure 9, the Sn contents deposited at sputtering powers of 0 W, 10 W, 20 W, and 30 W were 0 at%, 2.1 at%, 3.6 at%, and 4.1 at%, respectively.

### 3.2. Electrical Properties of Sn-Doped Ga_2_O_3_ Thin Films

**Figure 10:**
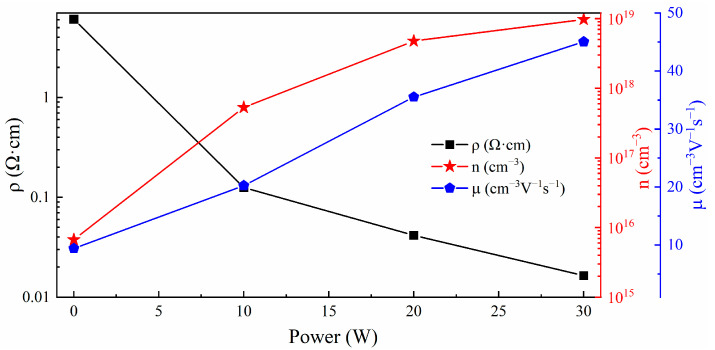
Electrical properties of thin films under different SnO_2_ sputtering powers.

From Figure 10, it can be seen that as the sputtering power of SnO_2_ target material changes in the range of 0~30 W, the resistivity of the Ga_2_O_3_ film gradually decreases from 6.08 × 10^−2^ Ω·cm to 0.016 × 10^−2^ Ω·cm, the Hall mobility increases from 9.39 cm^3^/Vs to 45.05 cm^3^/Vs, and the carrier concentration increases from 0.0067 × 10^18^ cm^−3^ to 9.72 × 10^18^ cm^−3^. The main reason for this electrical pattern change is that the Sn content in the thin film increases with the increase in the sputtering power of the SnO_2_ target material. Sn^4+^ can replace atoms occupying the position of Ga^3+^ in the lattice or exist as interstitial atoms in the lattice. In both states, Sn generates electrons due to the ionization of valence electrons, which increases the carrier concentration of a Ga_2_O_3_ thin film and reduces the film resistivity. At the same time, the reduction in bandgap width in Sn-doped Ga_2_O_3_ thin films is also beneficial for the inter-band transport of charge carriers.

### 3.3. Optical Performance of Sn-Doped Ga_2_O_3_ Thin Films

**Figure 11:**
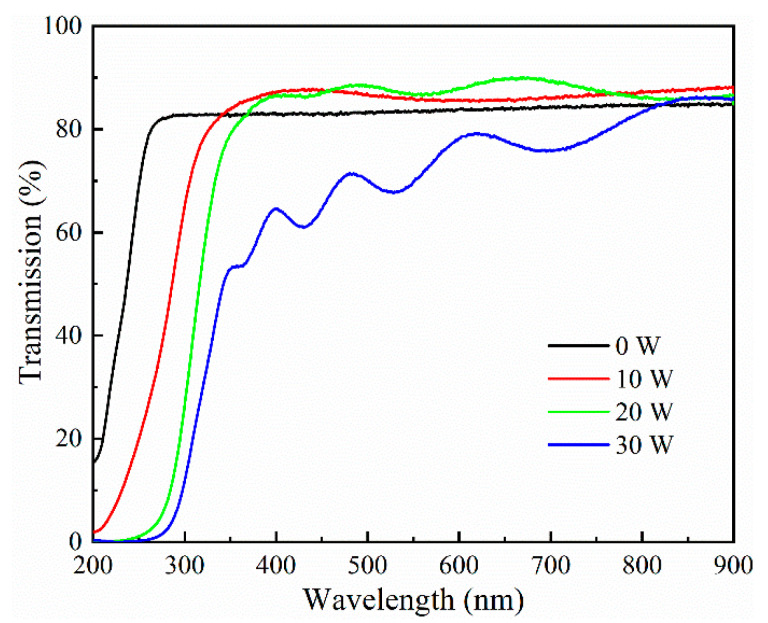
Transmittance curves of Sn-doped Ga_2_O_3_ thin films.

**Figure 12:**
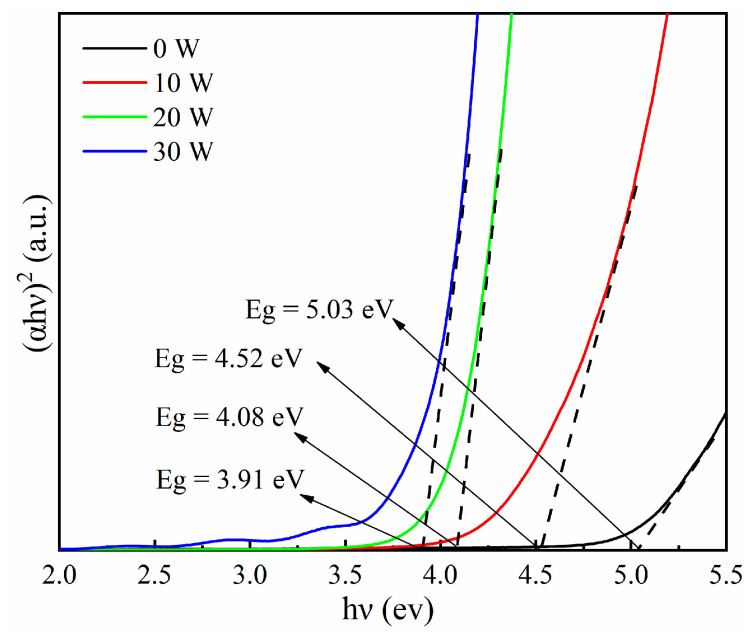
Optical bandgap of Sn-doped Ga_2_O_3_ thin films.

Figure 11 shows the transmittance curves of Sn-doped Ga_2_O_3_ thin films with different target sputtering powers: 0 W, 10 W, 20 W, and 30 W. The analysis results show that the thin films grown under different sputtering powers of the SnO_2_ target materials have steep absorption edges in the ultraviolet band and that interference fringes can be seen in the transmission spectrum in the visible light band range. This indicates that Ga_2_O_3_ thin films with different Sn-doping contents have good thickness uniformity and density. At the same time, the average transmittance of the thin film in the visible light region under a sputtering power of 0~20 W from the SnO_2_ target material is found to be greater than 82%. Additionally, with the increase in Sn-doping content, when the sputtering power of the SnO_2_ target material increases to 30 W, the average transmittance of the Sn-doped Ga_2_O_3_ film in the visible light region significantly decreases. This may be due to the increase in the number of defects in the film after the Sn doping content reaches a certain value, which leads to an increase in the light absorption coefficient of the film and a decrease in the average transmittance of the film in the visible light range [35]. With the increase in Sn-doping content, the film exhibits clear and single absorption edges in the range of 200~350 nm, and with the increase in Sn-doping content, the absorption edge of the film undergoes a red shift, which is attributed to the bandgap-narrowing effect [25]. The optical bandgap can be calculated using the following equation [36]:

(1) $${\left(\alpha h\nu \right)}^{2}=A\left(h\nu -E_{g}\right)$$

where *α* is the light absorption rate of the Ga_2_O_3_ thin film, *hv* is the photon energy, *A* is the proportional constant, and *E_g_* is the thin film optical bandgap. The relationship curve between *hv* and (*αhv*)^2^ is shown in Figure 12. The bandgap width of Ga_2_O_3_ thin films can be seen to gradually decrease from 5.03 eV to 3.91 eV with the increase in Sn-doping content. Furthermore, the content of Sn^4+^ as a donor impurity increases with the increase in doping concentration, the bandgap width of Ga_2_O_3_ thin films decreases due to the bandgap narrowing effect [37].

### 3.4. Performance of Sn-Doped Ga_2_O_3_ Thin Film MSM Ultraviolet Detector

**Figure 13:**
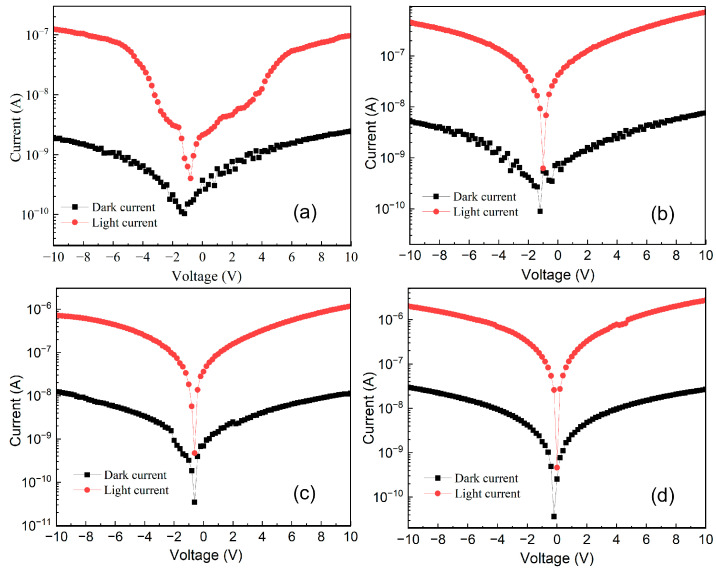
Dark and light current curves of detectors under different sputtering powers of SnO_2_ target materials, (**a**) 0 W, (**b**) 10 W, (**c**) 20 W, (**d**) 30 W.

MSM-type ultraviolet detectors were prepared for the Sn-doped Ga_2_O_3_ thin films and undoped Ga_2_O_3_ thin films, respectively. Figure 13 displays the dark current curves of the detectors. At a bias voltage of 10 V, the sputtering powers of the SnO_2_ target material are 0 W, 10 W, 20 W, and 30 W, and the corresponding dark currents of the device are 2.46 nA, 7.6 nA, 11.48 nA, and 26.74 nA, respectively. With the increase in Sn-doping content in the Ga_2_O_3_ film, more Sn atoms act as effective donor impurities to provide electrons for the Ga_2_O_3_ film, thereby increasing the carrier concentration in the film. As the Sn-doping content increases to 30 W, the *I–V* characteristic curve of the device under no ultraviolet light gradually shows a linear trend. As the Sn doping content increases, the carrier concentration in the film increases, and a decrease in the work function of Ga_2_O_3_ results in a thinning of the potential barrier between the semiconductor and the metal [38] and an enhancement in the carrier tunneling effect, leading to gradual ohmic contact in the device’s contact characteristics. Under 254 nm, 100 μW/cm^2^ ultraviolet light irradiation, the *I–V* characteristic curves of different Sn-doped detectors show that compared with undoped devices, at a bias voltage of 10 V, the sputtering powers of the SnO_2_ target material are 0 W, 10 W, 20 W, and 30 W, and the corresponding light currents of the device are 0.09 μA, 0.72 μA, 1.17 μA, and 2.72 μA, respectively. The photocurrent of Sn-doped devices increases by two orders of magnitude compared with their dark current.

**Figure 14:**
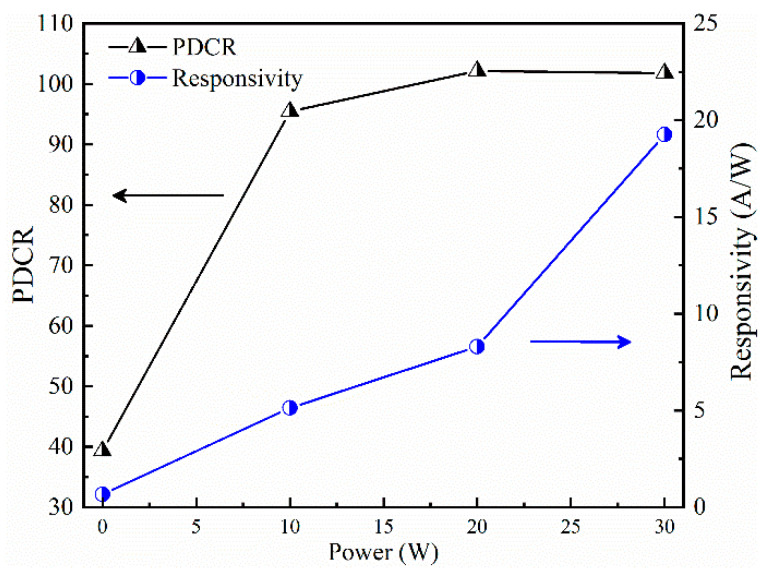
The relationship between the light-to-dark current ratio and responsiveness of detectors and the sputtering power of SnO_2_ target materials.

To further analyze the effect of Sn-doping content on the responsivity (*R*) and photo dark current ratio (PDCR) of Sn-doped Ga_2_O_3_-based ultraviolet detectors under external light signals, Formulas (2) and (3) [39] were used to calculate the *R* and *I_p_*/*I_d_* of undoped devices and devices prepared under different SnO_2_ target sputtering powers under an external bias voltage of 10 V. The calculation results of *R* and *I_p_*/*I_d_* are shown in Figure 14.

(2) $$PDCR=\frac{I_{light}-I_{dark}}{I_{dark}}$$

(3) $$R=\frac{{(I}_{light}-I_{dark})}{P_{\lambda }S}$$

The *R* values of the undoped devices and SnO_2_ target materials prepared at sputtering powers of 10 W, 20 W, and 30 W under 10 V bias were 0.67 A/W, 5.13 A/W, 8.3 A/W, and 19.25 A/W, respectively, with *I_p_*/*I_d_* values of 39, 95, 102, and 101. Under a bias voltage of 10 V, the *R* of devices prepared with a SnO_2_ target sputtering power of 30 W increased by approximately 54 times compared with the undoped devices, and the *I_p_*/*I_d_* increased by approximately 5.5 times. The increase in responsiveness and photoelectric current ratio can be attributed to the improvement in film conductivity and the introduction of impurity levels, which increased the internal gain of the film. Therefore, increasing the Sn-doping content in Ga_2_O_3_ thin films can effectively improve the photoelectric conversion ability of ultraviolet photodetectors to external light signals while ensuring a strong photo-to-dark current ratio.

**Figure 15:**
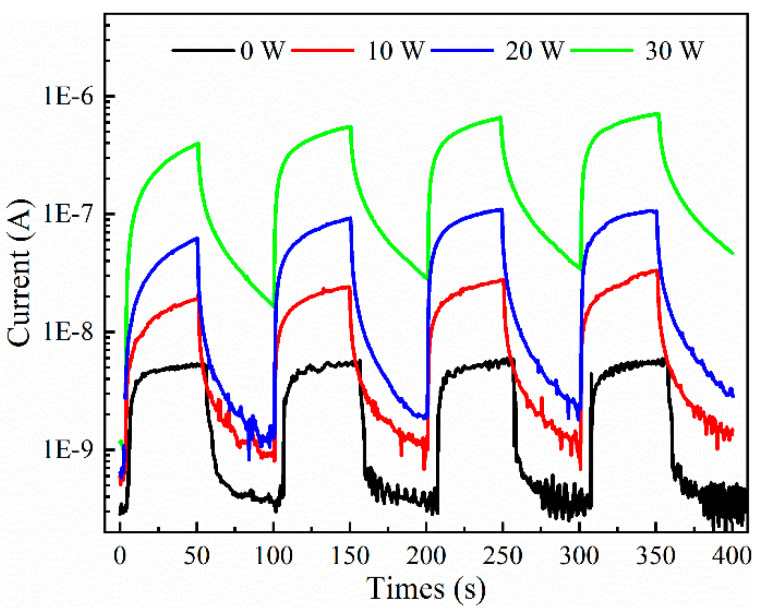
I-t characteristic curves of detector response to 254 nm light under different sputtering powers of SnO_2_ target materials.

**Figure 16:**
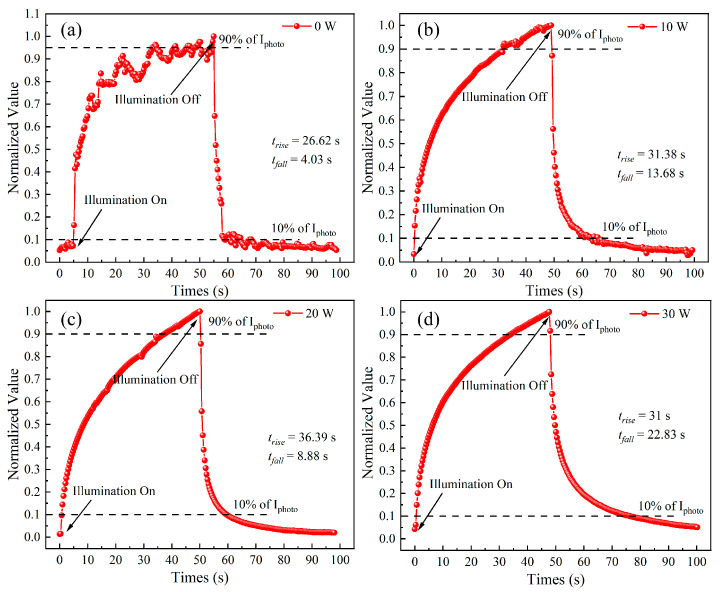
Individual on/off cycling of I-t characteristic curves of detector response. (**a**) 0 W, (**b**) 10 W, (**c**) 20 W, (**d**) 30 W.

Figure 15 shows the transient response characteristic curve under 100 μW/cm^2^ ultraviolet light irradiation for the MSM-type ultraviolet detector prepared from the Sn-doped Ga_2_O_3_ thin films deposited at different sputtering powers of SnO_2_ target materials at 254 nm and 100 nm, where the switching time of the ultraviolet light source is 50 s each. As shown in the figure, the device exhibits good stability and repeatability in response to 254 nm ultraviolet light in multiple switching cycles. The I-t characteristic curves of the devices prepared with SnO_2_ target sputtering powers of 0 W, 10 W, 20 W, and 30 W were normalized for a single cycle, as shown in Figure 16. The rise time *t_rise_* and recovery time *t_fall_* of the detector were calculated by normalizing the curve. As the sputtering power of the SnO_2_ target increased, the detector at 20 W reached the maximum rise time (36.39 s) and *t_fall_* increased overall. Compared with undoped Ga_2_O_3_, the recovery time of the Sn-doped devices significantly increased. The recovery time of the MSM-type detectors consists of two parts: fast response and slow response. A fast response is formed by changes in the carrier concentration inside the thin film caused by a change in the light source, while the slow response is due to defects inside the deposited thin film. The recovery time of doped devices increases with the increase in sputtering power of the SnO_2_ target material, which can be attributed to the change in the crystal quality of the thin film. The doping of Sn atoms introduces a large number of defects into Ga_2_O_3_ thin films, which act as traps to hinder the recombination of photo-generated carriers after light removal [40], leading to an extension of the device recovery time.

**Figure 17:**
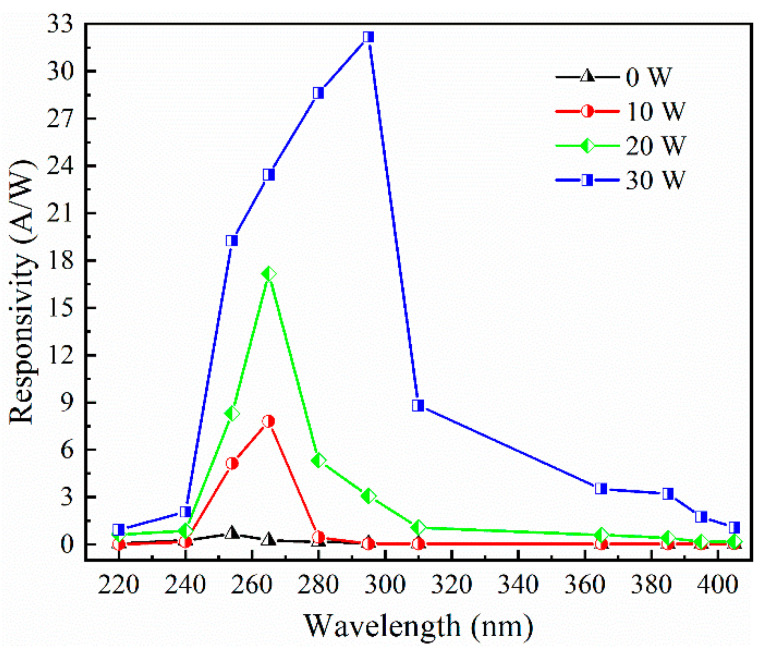
Spectral response characteristic curves of SnO_2_ target materials under different sputtering powers.

Figure 17 shows the spectral response characteristics of Sn-doped Ga_2_O_3_ ultraviolet detectors under different sputtering powers of the SnO_2_ target materials at a bias voltage of 10 V, and make the light intensity 100 μW/cm^2^ at different wavelengths. In the daily blind ultraviolet region of 200–275 nm, the photoresponsivity of Sn-doped Ga_2_O_3_-based ultraviolet detectors is much higher than that of undoped devices. The undoped device reached its peak UV responsivity near 254 nm, and the responsivity value is 0.67 A/W at 254 nm. As the sputtering power of the SnO_2_ target increased, the peak spectral response of the detector shifted towards longer wavelengths, resulting in a red shift in the detector’s spectral response. The peak wavelength of the detector’s responsivity increased from 254 nm for undoped detectors to 295 nm for SnO_2_ targets, with a sputtering power of 30 W, and the values of responsivity were 32.15 A/W at 295 nm and 19.25 A/W at 254 nm. Doping can effectively broaden the spectral response wavelength of Ga_2_O_3_-based UV photodetectors. The spectral response red shift of Ga_2_O_3_-based ultraviolet detectors is mainly due to the introduction of impurity energy levels from Sn doping in Ga_2_O_3_ films, and the bandgap width of SnO_2_ is 3.6 eV, which is smaller than that of Ga_2_O_3_. Therefore, Sn doping in Ga_2_O_3_ films can effectively reduce the bandgap of Ga_2_O_3_, thereby causing the spectral response cutoff wavelength of Ga_2_O_3_-based ultraviolet detectors to shift towards longer wavelengths.

## 4. Conclusions

In summary, we have investigated the structural, composition, electrical, and optical properties of Sn-doped Ga_2_O_3_ thin films and systematically characterized the response characteristic of MSM ultraviolet detectors with magnetron co-sputtering. The results reveal that all the prepared films were monoclinic β-Ga_2_O_3_ films, Sn doping can effectively change the crystalline quality of the thin films, and the Sn content can reach 4.1 at% under 30 W DC power. A maximum carrier concentration of 9.72 × 10^18^ cm^−3^ and a minimum resistivity of 0.016 × 10^−2^ Ω·cm were obtained at 30 W. The results of the optical performance and optical bandgap indicate that the average transmittance of thin film samples prepared under a sputtering power of 0~20 W for a SnO_2_ target material in the visible light region is greater than 82% and that the bandgap width of Ga_2_O_3_ thin films gradually decreases from 5.03 eV to 3.91 eV with increasing Sn-doping power. From the dark and light *I–V* characteristics of the Ga_2_O_3_ detector under different Sn-doped sputtering powers, the values of R and PDCR were found to reach 19.25 A/W and 101 under 30 W DC power. Time responses of *t_rise_* = 31 s and *t_fall_* = 22.83 s were obtained. A maximum spectral responsivity of 32.15 A/W was obtained at 295 nm.

## Data Availability

The original contributions presented in the study are included in the article material, further inquiries can be directed to the corresponding author.

## References

[1] Chen H., Liu K., Hu L., Al-Ghamdi A.A., Fang X. (2015). New concept ultraviolet photodetectors. Mater. Today.

[2] Li Z., Li Z., Shi Z., Zhu P., Wang Z., Zhang J., Li Y., He P., Zhang S. (2023). ALD prepared silver nanowire/ZnO thin film for ultraviolet detectors. Mater. Today Commun..

[3] Xin Y., Zhang W., Gao Z., Xiu J., Yu D., Li Z., Zhu M. (2024). Preparation of ZnGa_2_O_4_-based deep ultraviolet photodetector with high photodetectivity by magnetron sputtering. Vacuum.

[4] Xu J., Zheng W., Huang F. (2019). Gallium oxide solar-blind ultraviolet photodetectors: A review. J. Mater. Chem. C.

[5] Senthilkumar L., Raja S., Babu R., Vasuki G. (2022). Enhanced electrical and optoelectronic properties of W doped SnO_2_ thin films. Opt. Mater..

[6] Dong L., Yu J., Zhang Y., Jia R. (2019). Elements (Si, Sn, and Mg) doped α-Ga_2_O_3_: First-principles investigations and predictions. Comput. Mater. Sci..

[7] Ken G., Keita K., Hisashi M., Yoshinao K., Bo M., Masataka H., Akito K., Shigenobu Y. (2018). Halide vapor phase epitaxy of Si doped β-Ga_2_O_3_ and its electrical properties. Thin Solid. Film..

[8] Rekha P., Gopalakrishnan N. (2021). Room temperature ammonia sensing performances of pure and Sn doped β-Ga_2_O_3_. Mater. Sci. Semicond. Process.

[9] Wang D., Ma X., Chen R., Le Y., Zhang B., Xiao H., Luan C., Ma J. (2022). Solar-blind ultraviolet photodetectors based on Ta-doped β-Ga_2_O_3_ heteroepitaxial films. Opt. Mater..

[10] Esmat F., Elaheh A., James S., Aaron R., Steven A. (2018). Deep level defects in Ge-doped (010) β-Ga_2_O_3_ layers grown by plasma-assisted molecular beam epitaxy. J. Appl. Phys..

[11] Wang D., Ge K., Meng D., Chen Z. (2023). P-type β-Ga_2_O_3_ films were prepared by Zn-doping using RF magnetron sputtering. Mater. Lett..

[12] Chu S.Y., Yeh T.H., Lee C.T., Lee H.Y. (2022). Mg-doped beta-Ga_2_O_3_ films deposited by plasma-enhanced atomic layer deposition system for metal-semiconductor-metal ultraviolet photodetectors. Mater. Sci. Semicond. Process.

[13] Zhang Q., Deng J., Li R. (2024). Study on the structural, optical and electrical properties of N-doped Ga_2_O_3_ films synthesized by sol-gel method. Mater. Sci. Semicond. Process.

[14] Zhang H., Deng J., Zhang Q., Wang X., Meng J., Xu Z., Li R., Zhang X., Zhang J. (2021). Trace amount of niobium doped β-Ga_2_O_3_ deep ultraviolet photodetector with enhanced photo-response. Optik.

[15] Wang C., Zhang Y.C., Fan W.H., Wu W.Y., Wuu D.S., Lien S.Y., Zhu W.Z. (2022). Texture evolution mechanism of pulsed laser deposited in-doped Ga_2_O_3_ film affected by laser fluence and its application in solar-blind photodetector. Vacuum.

[16] Lin T., Xie C., Yang S., Xie J., Liu Y., Chen S., Huang H., Dang J., Huang R., Duan Y. (2024). Investigation on the Bandgap-Adjustable (Ga_1−x_In_x_)_2_O_3_ Film Prepared by Magnetron Sputtering. ACS Appl. Electron. Mater..

[17] Shang Y., Tang K., Chen Z., Zhang Z., Deng J., Hu Y., Gu K., Cao M., Wang L., Huang J. (2021). Growth and characterization of Ta-doped Ga_2_O_3_ films deposited by magnetron sputtering. Mater. Sci. Semicond. Process.

[18] Cui H., Mohamed H.F., Xia C., Sai Q., Zhou W., Qi H., Zhao J., Si J., Ji X. (2019). Tuning electrical conductivity of β-Ga_2_O_3_ single crystals by Ta doping. J. Alloys Compd..

[19] Mi W., Li Z., Luan C., Xiao H., Zhao C., Ma J. (2015). Transparent conducting tin-doped Ga_2_O_3_ films deposited on MgAl_2_O_4_ (100) substrates by MOCVD. Ceram. Int..

[20] Zhang Y., Yan J., Zhao G., Xie W. (2010). First-principles study on electronic structure and optical properties of Sn-doped β-Ga_2_O_3_. Phys. B.

[21] Fan M.M., Lu Y.J., Xu K.L., Cui Y.X., Cao L., Li X.Y. (2020). Growth and characterization of Sn-doped β-Ga_2_O_3_ thin films by chemical vapor deposition using solid powder precursors toward solar-blind ultraviolet photodetection. Appl. Surf. Sci..

[22] Zhu X., Wu Y., Li G., Lu W. (2023). In-situ composition development of Zn/In-doped Ga_2_O_3_ nanowire with ultrahigh responsivity and long-term stability for deep-UV photodetector. J. Alloys Compd..

[23] Li J., Wang Y., Li W., Zhang T., Tian X., Zhang Y., Feng Q., Zhang J., Hao Y. (2024). Effect of tin source temperature on the β-Ga_2_O_3_ film deposited by MOCVD. J. Mater. Sci. Eng. B.

[24] Mauze A., Zhang Y., Itoh T., Ahmadi E., Speck J.S. (2020). Speck. Sn doping of (010) β-Ga_2_O_3_ films grown by plasma-assisted molecular beam epitaxy. Appl. Phys. Lett..

[25] Jiang F., Huang M., Chen Z., Zhang Y., He Y., Zhang Q. (2023). High-performance (Ga,Sn)O_3_-based self-powered solar-blind photodetectors achieved via the sol-gel technique and modulating carrier concentrations. Sens. Actuators A.

[26] Fan Z.Y., Yang M.J., Fan B.Y., Mavrič A., Pastukhova N., Valant M., Li B.-L., Feng K., Liu D.-L., Deng G.-W. (2022). Plasma-enhanced atomic layer deposition of amorphous Ga_2_O_3_ for solar-blind photodetection. J. Electron. Sci. Technol..

[27] Yadav M.K., Mondal A., Das S., Sharma S.K., Bag A. (2020). Impact of annealing temperature on band-alignment of PLD grown Ga_2_O_3_/Si (100) heterointerface. J. Alloys Compd..

[28] Wang C., Fan W.H., Zhang Y.C., Kang P.C., Wu W.Y., Wuu D.S., Lien S.-Y., Zhu W.Z. (2023). Effect of oxygen flow ratio on the performance of RF magnetron sputtered Sn-doped Ga_2_O_3_ films and ultraviolet photodetector. Ceram. Int..

[29] Yu J., Nie Z., Dong L., Yuan L., Li D., Huang Y., Zhang L., Zhang Y., Jia R. (2019). Influence of annealing temperature on structure and photoelectrical performance of β-Ga_2_O_3_/4H-SiC heterojunction photodetectors. J. Alloys Compd..

[30] Jiao T., Dang X., Chen W., Li Z., Diao Z., Chen P., Dong X., Zhang Y., Zhang B. (2023). Self-powered Schottky barrier photodiodes based on homoepitaxial Ga_2_O_3_ film. Mater. Lett..

[31] Li M.Q., Yang N., Wang G.G., Zhang H.Y., Han J.C. (2019). Highly preferred orientation of Ga_2_O_3_ films sputtered on SiC substrates for deep UV photodetector application. Appl. Surf. Sci..

[32] Singh A.K., Yen C.C., Chang K.P., Wuu D.S. (2023). Structural and photoluminescence properties of Co-Sputtered p-type Zn-doped β-Ga_2_O_3_ thin films on sapphire substrates. J. Lumin..

[33] Pandeeswari R., Jeyaprakash B., Pandiyarasan V., Balamurugan D. (2022). Enhanced selective ammonia detection of spray deposited Cd-doped β-Ga_2_O_3_ thin films with low hysteresis effect. Ceram. Int..

[34] Pramod M., Shiv K., Anand P., Lalit K., Arnab M., Ankush B. (2024). Unveiling structural and optical properties of Sn-doped β-Ga_2_O_3_:A correlation of experimental and theoretical observations. Mater. Sci. Eng. B.

[35] Ravichandran K., Thirumurugan K., Begum N.J., Snega S. (2013). Investigation of p-type SnO_2_:Zn films deposited using a simplified spray pyrolysis technique. Superlattices Microstruct..

[36] Miao W., Li X., Zhang Q. (2006). Transparent conductive In_2_O_3_:Mo thin films prepared by reactive direct current magnetron sputtering at room temperature. Thin Solid. Film..

[37] Choi Y., Lee S.S., Lee W.J., Park I.K. (2023). Zn-doping induced phase control mechanism of Ga_2_O_3_ thin films by spray pyrolysis deposition for application of solar-blind ultraviolet photodetector. Mater. Today Chem..

[38] Shi Y., Meng J., Chen J., Wu R., Zhang L., Jiang J., Deng J., Yin Z., Zhang X. (2024). Enhanced electrical conductivity and reduced work function of β-Ga_2_O_3_ thin films by hydrogen plasma treatment. J. Alloys Compd..

[39] Zhang M., Ma W., Liu Z., Yang L., Li S., Guo Y., Tang W. (2023). Sensing performance of β-Ga_2_O_3_ metal-semiconductor-metal deep ultraviolet photodetectors with refractory TiW electrodes at high temperatures. Results Phys..

[40] Pu S., Ou Y., Cai M.Q., Wu Z. (2023). High-sensitivity Solar-blind photodetector based on Ga_2_O_3_ films through manipulating oxygen vacancies. Mater. Lett..

